# Diagnostic Challenges in Lymphangioleiomyomatosis: From Ovarian Mass to Systemic Diagnosis

**DOI:** 10.15388/Amed.2025.32.1.19

**Published:** 2025-02-18

**Authors:** Neringa Jansevičiūtė, Linas Andreika

**Affiliations:** 1 Faculty of Medicine, Vilnius University, Vilnius, Lithuania; 2 Clinic of Obstetrics and Gynaecology, Institute of Clinical Medicine, Vilnius University, Vilnius, Lithuania

**Keywords:** lymphangioleiomyomatosis, ovarian mass, extrapulmonary manifestation, limfangiolejomiomatozė, kiaušidžių navikas, ekstrapulmoninė manifestacija

## Abstract

**Background:**

*Lymphangioleiomyomatosis* (LAM) is a rare neoplastic disorder characterized by the proliferation of atypical smooth muscle-like or epithelioid cells within the lungs and axial lymphatic system. This pathological process leads to the formation of pulmonary cysts and impaired respiratory function. Although the disease primarily involves the lungs, extrapulmonary manifestations can occur in the abdominal cavity, lymphatic system, and retroperitoneum.

**Clinical case:**

A 48-year-old woman presented with abdominal numbness, leading to the discovery of a right ovarian mass. CT and MRI identified non-malignant solid mass in the ovary, as well as thin-walled cysts in the lungs, retroperitoneal pelvis, and upper abdomen, suggesting LAM. The patient was referred for pulmonology evaluation. Genetic testing and lung biopsy were inconclusive. One year later, during laparoscopic hysterectomy for early-stage uterine cancer, a biopsy of a left iliac lesion confirmed LAM. Postoperatively, the patient developed lymphocytic and chylous ascites, requiring further surgical intervention. However, the ascites recurred, and it was managed with diuretic therapy. Following the confirmed diagnosis, *Sirolimus* therapy was initiated. To date, the patient has not exhibited any significant respiratory symptoms, and follow-up lung imaging has shown no evidence of disease progression.

**Conclusions:**

Due to its rarity, diverse symptoms, and involvement of multiple organs, diagnosis of LAM is challenging. It requires careful clinical observation and a multidisciplinary approach. Early and accurate diagnosis, combined with timely therapeutic interventions, has the potential to significantly improve the patient outcomes in LAM.

## Abstract (it)

**Įvadas:**

Limfangiolejomiomatozė (LAM) yra reta neoplastinė liga, kuriai būdinga netipinių lygiųjų raumenų arba epitelinių ląstelių proliferacija plaučiuose ir ašinėje limfinėje sistemoje. Šis patologinis procesas lemia cistų formavimąsi plaučiuose, dėl jų sutrikdoma kvėpavimo funkcija. Nors liga dažniausiai pažeidžia plaučius, taip pat galima jos ekstrapulmoninė raiška, sukelianti limfinės sistemos, retroperitoninės ar pilvo srities pažeidimus.

**Klinikinis atvejis:**

48 metų moteris kreipėsi dėl pilvo srities tirpimo, echoskopiškai diagnozuotas dešiniosios kiaušidės darinys. Detalesnio ištyrimo metu atlikta KT ir MRT patvirtino darinį kiaušidėje. Vaizdiniuose tyrimuose taip pat matomos plonasienės cistos plaučiuose, retroperitoninėje dubens srityje ir viršutinėje pilvo dalyje, tai leido įtarti LAM. Pacientė buvo siųsta pulmonologo konsultacijai. Genetiniai tyrimai ir plaučių biopsija nepatvirtino diagnozės. Po metų, atliekant laparoskopinę histerektomiją dėl ankstyvos stadijos gimdos vėžio, buvo paimta biopsinė medžiaga iš kairės klubinės srities, tai leido histologiškai patvirtinti LAM diagnozę. Po operacijos pacientei išsivystė chiliozinis ascitas, dėl neefektyvaus drenavimo gydytas chirurginiu būdu. Po kelių savaičių ascitas atsinaujino, gydymui buvo skirti diuretikai, gydymo efektas buvo teigiamas. Histologiškai patvirtinus diagnozę, pradėtas gydymas sirolimu. Pacientės plaučių funkcija išlieka gera, plaučių vaizdiniai tyrimai nerodo ligos progresavimo.

**Išvados:**

LAM kelia diagnostinių sunkumų dėl jos retumo, įvairialypių simptomų ir daugelio organų pažeidimo. Ligos diagnozei reikia atidaus klinikinio vertinimo ir multidisciplininio požiūrio. Ankstyva ir tiksli diagnozė bei laiku pritaikytas specifinis gydymas gali reikšmingai pagerinti pacientų, sergančių LAM, prognozę.

## Introduction

*Lymphangioleiomyomatosis* (LAM) is a rare, metastasizing neoplastic disease which primarily affects women of reproductive age [1,2]. Sixteen out of every million reproductive age women are estimated to have LAM [3]. Female hormones, particularly estrogens, significantly influence its course [4]. LAM may present sporadically (S-LAM) or in association with tuberous sclerosis complex (TSC-LAM), which is caused by mutations in the TSC1 and TSC2 tumor suppressor genes [5,6].

LAM is characterized by an abnormal proliferation of smooth muscle-like cells which commonly infiltrate the lungs and the axial lymphatic system, leading to pulmonary cysts and progressive respiratory impairment [2,7]. Although the disease most often presents with progressive dyspnea on exertion, cough, chest pain, and recurrent pneumothorax, depending on the organs involved, extrapulmonary manifestations may occur in the abdominal cavity, lymphatic system, and retroperitoneum [4,8,9]. Clinical features can include chylous pleural effusions or abdominal masses such as angiomyolipomas and lymphangioleiomyomas [2]. Pneumothorax serves as the initial clinical manifestation in approximately 40% of patients, and ultimately affects around 66% of individuals throughout the progression of the disease [10]. Because of its rarity, diverse symptoms, and involvement of multiple organs, diagnosis of LAM is challenging.

The aim of our article is to highlight the struggles of detection and confirmation of extrapulmonic LAM. We present a case report on rare manifestation of LAM which enabled early disease detection in an asymptomatic patient, allowing for timely initiation of specific treatment. This case highlights the importance of a comprehensive, multidisciplinary approach for the timely diagnosis and optimal treatment of LAM.

## Case report

A 48-year-old premenopause woman of average physique complained of abdominal numbness, leading to a pelvic ultrasound which revealed a solid mass in the right ovary. To assess the risk of ovarian tumor malignancy, CA 125 was tested and found to be within the normal range. Consequently, an MRI was recommended to differentiate between ovarian and retroperitoneal tumors. MRI confirmed a low risk (O-RADS 3) solid mass in the right ovary, which was histologically identified as a fibroma after excision during the laparoscopic right salpingoovarectomy. In addition, MRI raised suspicion of LAM in the left iliac region and identified a possible lymphatic venous malformation, requiring further investigation for a definitive diagnosis. A whole-body CT scan unveiled multiple round thin-walled cysts of varying sizes in the lungs, retroperitoneal pelvis, and upper abdomen. Radiological imaging suggested LAM. Metastatic diseases were considered unlikely due to the histological confirmation of a benign ovarian fibroma and its predominantly cystic, rather than solid, appearance on CT.

**Fig. 1:**
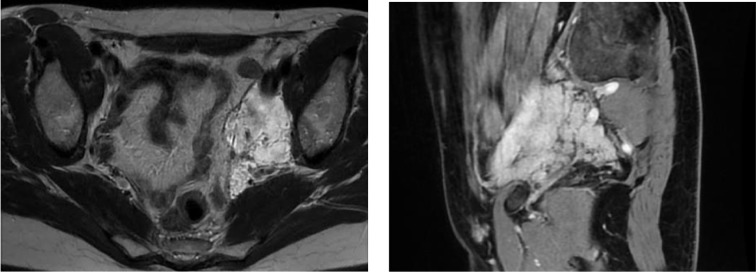
MRI: left iliac region. Suspicion of LAM

**Fig. 2:**
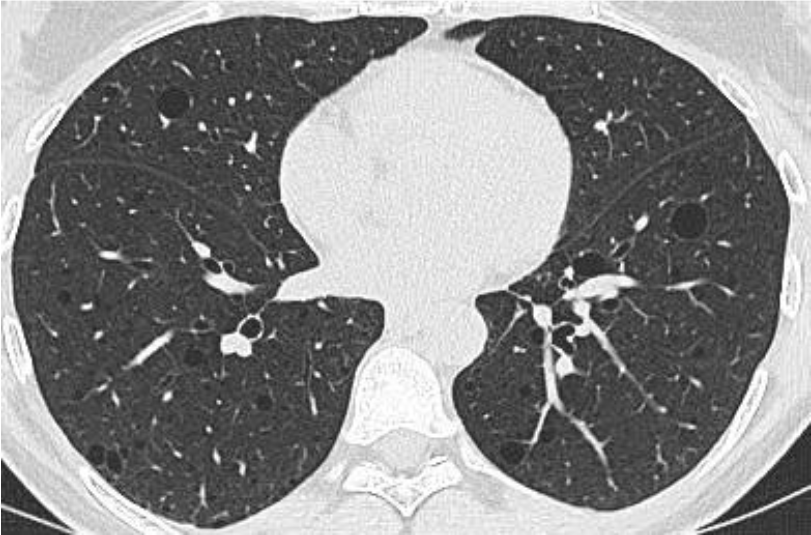
CT scan: multiple cysts in lungs

**Fig. 3:**
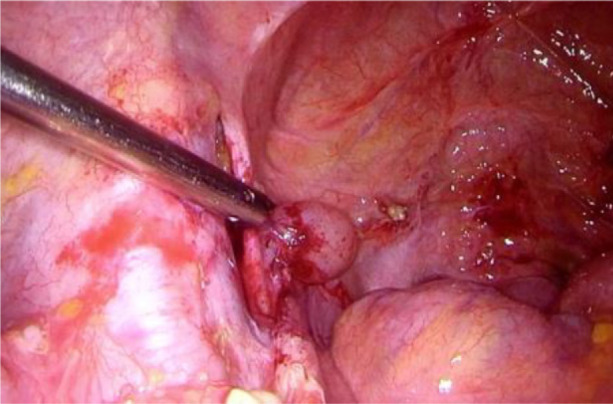
Left iliac lymphocyst repair

**Fig. 4:**
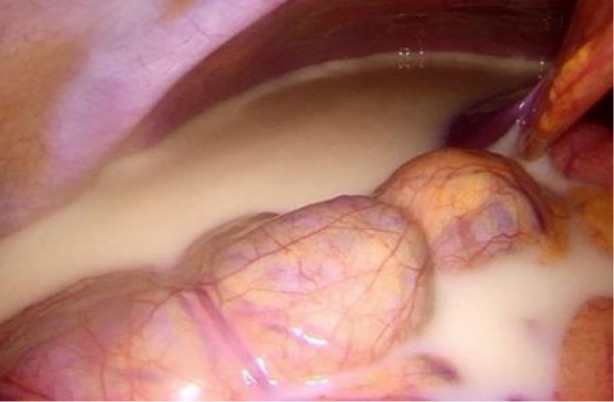
Chylous ascites

**Fig. 5:**
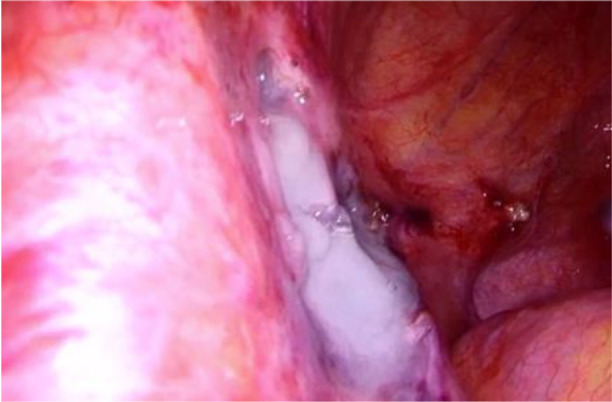
Hemostatic glue

The patient was referred to a pulmonologist for further evaluation. A lung tissue cryobiopsy, followed by a transbronchial biopsy, indicated nonspecific findings and failed to confirm the diagnosis. Genetic testing results indicated no pathogenic or likely pathogenic variants in the coding and surrounding regions of the TSC1 and TSC2 genes, which are linked to tuberous sclerosis and LAM. Since the performed tests did not confirm the diagnosis, an alternative method was needed to verify LAM. As the patient was newly diagnosed with uterine cancer, a laparoscopic hysterectomy, left salpingoovarectomy, and sentinel lymph node biopsy were required. This provided a great opportunity to perform a biopsy of the mass in the left iliac region. The biopsy successfully confirmed LAM.

Shortly after the procedure, the patient developed a lymphocyst and chylous ascites, necessitating surgical intervention due to the ineffectiveness of paracentesis. During the laparoscopic surgery, 4.5 liters of chylous ascites were drained, and a 2 cm cystic lesion was identified in the left iliac region at the site of the lymphangioleiomyoma, with active lymphatic leakage. The lymphocyst was fenestrated, coagulated using an ultrasound knife and covered with hemostatic glue.

However, ascites recurred two weeks later. Low doses of potassium-sparing diuretics were administered with notable effect. Specific treatment with *Sirolimus* was initiated after LAM was confirmed histologically.

The patient received treatment recommendations regarding the potential increased risk of pneumothorax and was advised to avoid estrogen-containing medications. After two months of *Sirolimus* therapy, pulmonary spirometry showed an FEV_1_ of 130%, with a mild gas diffusion impairment (DLCO adjusted to 69%). While on *Sirolimus*, the patient developed oral ulcers, which were treated with topical gel. Routine blood tests, including glucose, cholesterol, aspartate aminotransferase (AST), alanine aminotransferase (ALT), and creatinine, are conducted to monitor for potential adverse effects. In the conducted tests, dyslipidemia was observed, and it is being treated under the supervision of a family doctor. Regular follow-up includes *Sirolimus* blood concentration testing and chest CT scans. No pneumothorax episodes have occurred, with lung imaging showing no signs of disease progression.

## Discussion

Diagnosing extrapulmonary LAM can be challenging due to its rarity and atypical presentations, which often lead to misdiagnosis [8]. However, in the past decade, a better understanding of LAM pathophysiology has enhanced the ability of clinicians to recognize the disease.

In the case of LAM, nearly two-thirds of patients initially display progressively worsening shortness of breath [2]. Respiratory symptoms are the most common manifestation of LAM and are often misdiagnosed as asthma, emphysema, or *Chronic Obstructive Pulmonary Disease* (COPD) [10,11]. Approximately 25% of patients show additional respiratory symptoms, such as cough, infections, and chest pain [2]. Pneumothorax is the initial complication in almost half of LAM patients, and frequent recurrences of pneumothorax are highly suggestive of the disease [4]. Chylous pleural effusions, abdominal masses, such as angiomyolipomas and lymphangioleiomyomas, are also possible manifestations [2].

Clinical signs often emerge following pregnancy or during estrogen therapy, for example, while taking contraceptive pills [4]. In the observed case, our patient did not use contraceptive pills or hormone replacement therapy and had no other risk factors. Since LAM primarily affects women of a reproductive age, pregnancy is a major concern for LAM patients, with 25% reporting worsened respiratory symptoms [12]. Patients diagnosed with LAM experience higher rates of preterm births and miscarriages compared to those without the condition [13]. The increased estrogen levels during pregnancy may contribute to disease progression [7]. Additionally, treatment with *Sirolimus* must be discontinued 12 weeks prior to pregnancy, throughout pregnancy and breastfeeding, which can further exacerbate the symptoms of LAM [12].

## Diagnostic

A definitive diagnosis of LAM is established when characteristic lung cysts on CT imaging are accompanied by additional features such as renal angiomyolipomas, lymphangiomyomas, signs of tuberous sclerosis complex, chylous pleural or abdominal effusions, or confirmed lymph node involvement [4,9]. Characteristic findings in CT scans show lung cysts measuring 2 to 30 mm, appearing as hypodense areas scattered across both lungs. The cyst count varies from a few isolated lesions to near-total lung replacement [11,14]. LAM can also be diagnosed when characteristic lung cysts are histologically confirmed via transbronchial, thoracoscopic, or open lung biopsy to contain LAM cells immunoreactive with the monoclonal antibody HMB-45 [15].

Together with clinical and radiological findings, serum VEGF-D can serve as a diagnostic tool for LAM [11,15]. A level exceeding 800 pg/mL is both sensitive and specific for confirming the diagnosis without requiring additional biopsies. However, it may not be available in some countries, which limits its use in the clinical practice [11]. In our case, the disease was diagnosed through radiological imaging, as the transbronchial biopsy was non-informative. The presence of multiple (>10), diffuse, bilateral, uniform, round, thin-walled lung cysts, along with lymphangioleiomyoma and chylous effusion, met the diagnostic criteria for a definitive diagnosis of LAM [9]. Although a biopsy of the lymphangioleiomyoma in the iliac region confirmed the diagnosis, the invasive approach led to complications.

When diagnosing LAM, it is crucial to exclude other cystic interstitial lung diseases, such as Birt-Hogg-Dubé (BHD) syndrome, *Lymphocytic Interstitial Pneumonia* (LIP), amyloidosis, and *Pulmonary Langerhans Cell Histiocytosis* (PLCH). Cyst distribution aids differentiation: LAM shows diffuse involvement, whereas BHD and LIP typically affect the lower lungs. Elliptical or paramediastinal cysts suggest BHD, often accompanied by skin or renal findings, while LIP features ground-glass opacities and nodules which can progress to honeycombing. LAM is further supported by lymphangioleiomyoma and chylous effusion, which is absent in BHD and LIP. Pulmonary amyloidosis typically presents as nodules, with cystic forms being rare, and requires a biopsy for diagnosis. PLCH displays irregular cysts, predominantly in the mid and upper lungs. Evaluation of the cyst distribution, shape, and the associated findings helps distinguish LAM from these other diseases [16,17].

## Pathophysiology

Although the precise pathogenesis of LAM is not fully understood, growing research highlights certain molecular drivers. Abnormal smooth muscle-like cells of an uncertain origin circulate through blood and lymphatic vessels, eventually lodging in the lungs, causing parenchymal damage and cyst formation [2]. It is thought that LAM may originate from mesenchymal cells in the uterus or lungs [7]. LAM arises from mutations in the *Tuberous Sclerosis Complex* (TSC) genes, which encode the proteins hamartin and tuberin. It can occur as TSC-associated LAM (TSC-LAM), or sporadically (S-LAM) [2,3]. In TSC-LAM, mutations in the TSC1 and TSC2 tumour suppressor genes lead to the loss of hamartin or tuberin proteins [5]. Mutations in the hamartin tuberin complex hyperactivate the mTOR signaling pathway, driving abnormal smooth muscle-like LAM cell growth and proliferation [18]. LAM cells also express lymphangiogenic factors (VEGF-C and VEGF-D) which promote blood and lymphatic vessel formation, facilitating cell proliferation, migration, and metastatic spread [2,4]. In addition, LAM exhibits multiple cancer-like characteristics, including increased cell proliferation, resistance to apoptosis, elevated expression of the genes involved in metastasis, immune system evasion, and maintenance of cellular stemness driven by heightened mTOR activity [7]. The identification of mTOR hyperactivation as key to lymphangioleiomyomatosis pathogenesis has enabled the development of *Sirolimus*, an oral mTOR inhibitor, as an effective treatment [1].

The disease mostly affects women, likely due to estrogen’s role in LAM cell growth [2]. Symptoms tend to worsen during times of hormonal change, such as pregnancy, use of birth control pills, or menstruation, but often stabilize after the menopause. Estrogen can activate the mTOR pathway through signaling cascades, thereby promoting the proliferation of LAM cells [4]. In the presented case, the patient initially underwent a right salpingoovarectomy, followed by a hysterectomy and left salpingoovarectomy a year later due to uterine cancer, resulting in iatrogenic menopause. This menopausal state may have contributed to favorable treatment outcomes

## Prognosis

Prior to the development of mTOR inhibitors such as *Sirolimus*, patients with LAM typically experience a gradual decline in the lung function leading to death or lung transplantation [11]. However, the introduction of *Sirolimus* dramatically changed these outcomes by substantially slowing the decline in the lung function and disease progression, helping control extrapulmonary manifestations, and enhancing survival in patients [11,19]. Although LAM remains a progressive disease, treatment with mTOR inhibitors, particularly *Sirolimus*, has been shown to slow down the decline in the lung function, reduce respiratory impairment, improve the quality of life and physical performance, and lower the VEGF-D levels [2,4]. Following a LAM diagnosis, in patients who do not undergo lung transplantation, patients have median survival rates of 94% at five years, 85% at ten years, 75% at fifteen years, and 64% at twenty years [20]. Serum VEGF-D levels, recognized as a biomarker for LAM, correlate with the disease progression and the overall survival outcomes [19]. Several other factors are associated with an adverse prognosis in LAM, including the premenopausal status, progressive dyspnea at presentation, the need for supplementary oxygen, and an FEV_1_ below 70% of the predicted value at diagnosis. Conversely, an older age at diagnosis, postmenopausal status, and an FEV_1_ above 70% predicted value are linked to a more favorable prognosis [2].

## Conclusions

We report a case in which the diagnostic process of an ovarian mass led to the identification of an extrapulmonary manifestation of LAM. The rarity of the disease makes it difficult for physicians to identify potential LAM complications [14]. Identification of the complications caused by LAM is essential for ensuring the optimal patient care. Timely diagnosis and treatment are crucial in improving the LAM outcomes, as mTOR inhibitors represent a targeted therapeutic option.
